# Double layer graft technique for horizontal alveolar ridge augmentation with staged implant placement: radiographic histological and implant stability analysis–a case report

**DOI:** 10.1186/s12903-024-04416-1

**Published:** 2024-06-13

**Authors:** Leila Elraee, Suzan Seif Allah Ibrahim, Doaa Adel-Khattab

**Affiliations:** 1 Periodontology and Diagnosis Department, Assistant Lecturer of Oral medicine, Modern Science and Arts University, October, Egypt; 2 Periodontology and Diagnosis, Ain Shams University, Cairo, Egypt

## Abstract

**Introduction:**

Horizontal ridge augmentation of a deficient alveolar bone site is performed either simultaneously with implant placement or in a staged approach prior to implant insertion. There are several available strategies for the augmentation of alveolar ridge deficiencies, including guided bone regeneration (GBR) through the use of barrier membranes. The success of the GBR approach mainly depends on the exclusion of soft tissue cells during bone remodeling.

**Case presentation:**

A healthy 25-year-old male patient presented with a missing upper left central incisor after clinical and radiographic examination, the site showed a class III defect horizontal atrophy. The procedure performed was the horizontal alveolar ridge augmentation using resorbable pericardium membrane with double layer graft technique (DLT) where autogenous bone placed as a first layer of the graft followed by xenograft as a second layer, the membrane was fixed with titanium pins. A cone beam computed tomography (CBCT) was performed before, immediately and 6 month following the surgery. After 6 months during implant placement, a core biopsy specimen was retrieved, stored and prepared for histological evaluation, with assessment of primary implant stability. The radiographic analysis showed a horizontal width gain of about 4 mm, at 6 month following implant placement, the implant was successfully osteointegrated with stability assessment also done after 6 months from placement.

**Conclusion:**

DLT was successfully used for horizontal alveolar ridge augmentation, thus allowing a prosthetically driven implant placement. More cases assessing implant survival and success are needed to confirm the results of this case report.

## Background

It is well known that subsequent to tooth extraction, the alveolar ridge undergoes resorption and atrophy thus exhibiting a wide range of dimensional changes [1, 2]. Many surgical methods and materials have been developed to correct alveolar bone deficiency, autogenous onlay bone graft was mainly used for the improvement of horizontally and vertically shrunken alveolar ridge [3]. Autogenous bone is still considered as the standard for the augmentation of the atrophic implant bed [4]. Different bone substitute materials are available without limitation as alloplast, allograft and xenograft, and additional surgical risks may be eliminated by avoiding a second intervention. However, their use will be associated with additional costs, and apart from foreign body reactions, bone substitutes may show inadequate osteogenesis [5] or completely lack osteogenesis depending on the material selected [6]. Also extracted teeth are a recent treatment alternative to autogenous bone for grafting purposes in block and particulate forms [7, 8].A cross-sectional retrospective study demonstrated the safety of mandibular grafts that reported excellent results in terms of survival rates with minor complications regarding the donor site area and is associated with high implant success. The successful rate for this technique for horizontal and vertical ridge augmentation was up to 97.1% [9] It is also associated with varying degrees of morbidity at the second surgery donor site, limited quantity of intraoral grafts, and the high morbidity of bone harvesting from extraoral sites with the disadvantage of rapid resorption [10, 11].

GBR is a surgical technique that increases the width of alveolar ridge for implant placement using barrier membranes with or without bone substitutes [12]. Autogenous bone grafts are not only believed to be the gold standard in regenerative surgery but also display a relative fast resorption rate, however it has ostogensis, osteoionductive and osteoconductive properties. Allografts also are known to resorb quickly but less than the autogenous and may have some osteoinductive activity. On the other hand, xenografts seem to resorb very slowly and display an osteoconductive property [13]. Resorbable membranes especially collagen pericardium membranes that had been designed to slowly resorb over a period of time up to 6 months [14], provide a biocompatible barrier that will allow the grafted region to consolidate specially with the permeability of the collagen membrane that enrich the graft with blood supply from the periosteum, and they have shown better soft tissue compatibility compared with nonresorbable membranes [15]. The predictability of GBR is based on several principles, should be achieve to obtain successful and repetitive outcomes, “P-A-S-S” 4 key principles: Primary wound closure, angiogenesis, space creation and maintenance, and Stability of wound [16].

A pilot study evaluated the bone formation after DLT which was performed by placement of allograft for the first layer followed by xenograft for the second layer, radiographic findings showed alveolar ridge increase, the newly formed tissues consisted mostly of a variable amount of new trabecular bone, some loose connective tissue, blood vessels, and occasional inflammatory cells [17].The aim of this case report is to present the clinical, radiographic, histological analysis with addition of stability assessment of the implant following lateral alveolar ridge augmentation using DLT with different layering of the grafts where fist layer was autogenous bone that represent 50% of the graft and second layer was xenograft which represent other 50% of the graft.

### Clinical presentation and case management

**Fig. 1:**
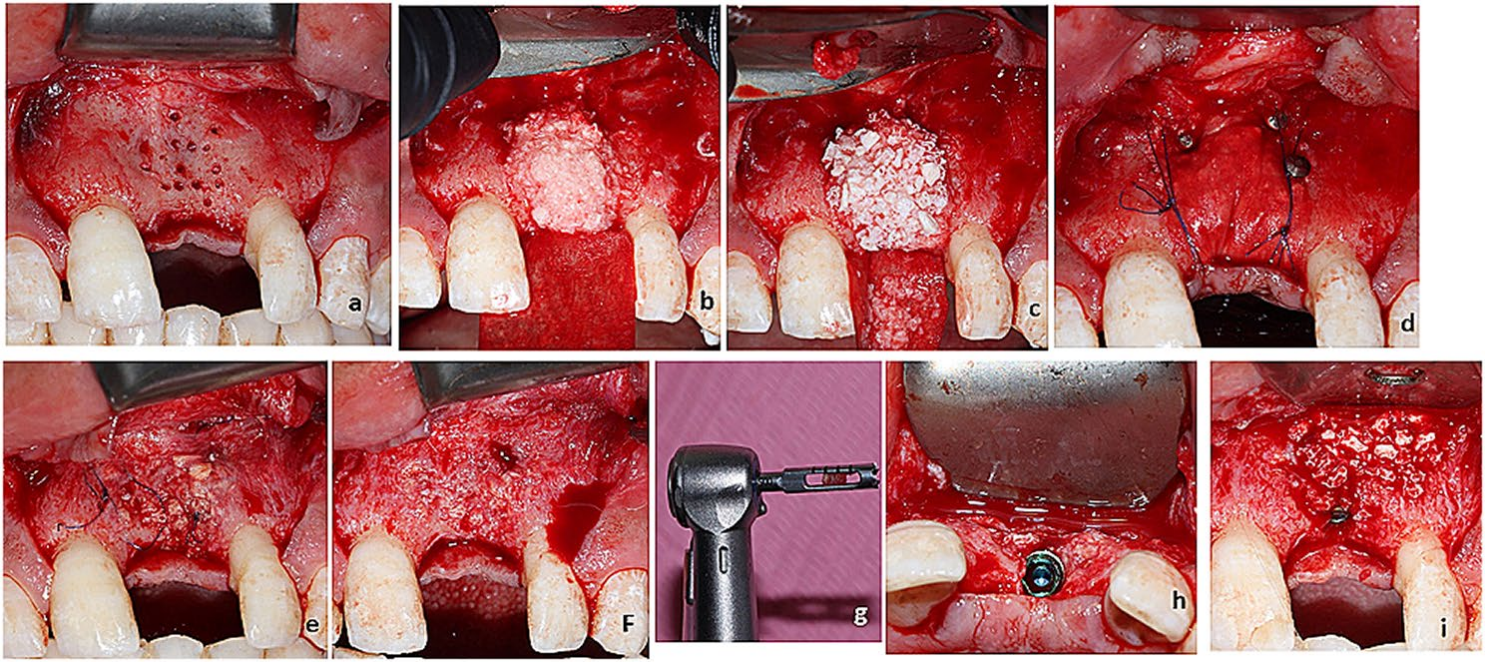
**a.** Frontal view for full thickness flap reflection & decortication, **b**: graft placement, first layer of autogenous bone, **c**: second layer of xenograft, **d**: frontal view for pericardium membrane fixation & periosteal suture over the membrane, **e**: Frontal view for full thickness flap reflection after 6 months from augmentation, **f, g**: core biopsy obtaining, **h**: implant placement, **i**: a layer of xenograft placed to act as veneering layer and also to fill the part where the biopsy taken

A healthy 25-years old male patient presented with a missing upper left central incisor (01) and he asked to restore it, after clinical and radiographic examination, the site showed a class III defect “horizontal atrophy” reference of alveolar ridge according to Tolstunov et al. [18], that need for hard tissue augmentation. The patient provided a written informed consent approved by Ain Shams University Ethical Committee (registry number FDASU-Rec ID 032148). The surgery was performed by the periodontist (LE), for the recipient site, a pyramidal full-thickness flap was elevated by making a crestal incision with two vertical anterior and posterior incisions that extended to the vestibule. The labial mucosa was reflected until 2 mm apical to the mucogingival junction. The recipient site was decorticated by a 0.8 mm bur to penetrate the underlying marrow and improve the blood supply to the graft (Fig. 1a). In the donor site, a full-thickness mucoperiosteal incision was made distal to the most posterior tooth in the mandible to the retromolar pad and ascending ramus. A submarginal incision was performed along the mucogingival line to facilitate the suturing procedure. A full-thickness mucoperiosteal flap was then reflected, harvesting particulate bone in both groups were performed using a cylindrical trephine-like drill (Ø: 4 mm) with a hollow part in the center and a stopper to limit the depth of drill penetration up to 4 mm by autogenous chip maker^1^. The autogenous bone chips then collected in a bone dish with sterial saline to avoid dehideration of the graft. Porcine pericardium membrane^2^ was adapted and fixed with fixation pins^3^ at the palatal aspect in both groups. For the D−group the sites was grafted with autogenous bone (first layer) which represent 50% of the graft and then a second layer deproteinized bovine bone mineral (DBBM)^4^ was placed on top of the autogenous graft. The fixed membrane was stretched enough to produce a balloon effect and fixed with titanium pins with a head diameter of 3 mm and a length of 3 mm and for more fixation of the barrier membrane, it was sutured with 5−0 non−resorbable Polypropylene monofilament suture^5^ (Fig. 1a−c).

The overlying flap was released by periosteal releasing incision before being sutured in a tension-free closure. Flap closure was accomplished through horizontal mattress and simple interrupted sutures using 5 − 0 polypropylene sutures for the crestal and releasing incisions.

### Postoperative care

Patient received antibiotics twice daily 1 g Augmentin^6^ for 7 days, 500−mg metronidazole^7^ three times per day for 7 days, anti−inflammatory^8^ tablets three times per day for 7 days and Ibuprofen^9^ 600 mg in case of pain. Patient was instructed to rinse his mouths twice daily with a 0.12% chlorherxidine digluconate^10^ mouth rinse and to avoid mechanical plaque removal at the site of surgery for 15–30 days. Sutures were removed 2 weeks after surgery. Six months following the healing, the patient underwent CBCT for graft site evaluation and to determine the dimensions of the implant to be placed. Ridge dimentional change was assessed clinically using bone caliper prior to the second surgery. A mucoperiosteal flap was elevated to expose the grafted area and a core biopsy sample with a 2 mm diameter was taken using a trephine bur^11^ 2 mm. After obtaining a core biopsy started with drilling for implant placement using sequential drills, an implant^12^ is placed and allowed for submerged healing (Fig. 1e−i).

Regarding the clinical evaluation, the ridge dimentions was assessed clinically by bone caliper preoperatively, immediately after the surgery and 6 months postoperatively. The measurement was performed with fixed points each time, the buccolingual width was measured at different levels. At the bone crest, 3 mm from the bone crest and 6 mm from the bone crest.

CBCT scans were performed using i-CAT Next Generation^14^ with exposure parameters of 85 KVp, 15 mA and 6 cm field of view (FOV). CBCT were obtained preoperatively, immediately postoperatively and 6 months postoperatively. The radiographs were analyzed using One− Viewer viewing software (iCATVision) and 0.2 mm of the CBCT slices thickness. The buccolingual width was measured into different levels. At the bone crest, 3 mm from the bone crest, and 6 mm from the bone crest. For standardization in the sagittal slice, the axial plane was adjusted to pass through the cemento enamel junction (CEJ) of the adjacent teeth. On the axial slice, the mesiodistal dimension from the distal surface to mesial surface of the adjeceent teeth was measured. The coronal plane was adjusted to be pass through the middle of the distance in order to be perpendicular to both buccal and lingual cortices. Measurement were all performed on the coronal slices.

### Clinical, radiographic and implant stability outcomes

**Fig. 2:**
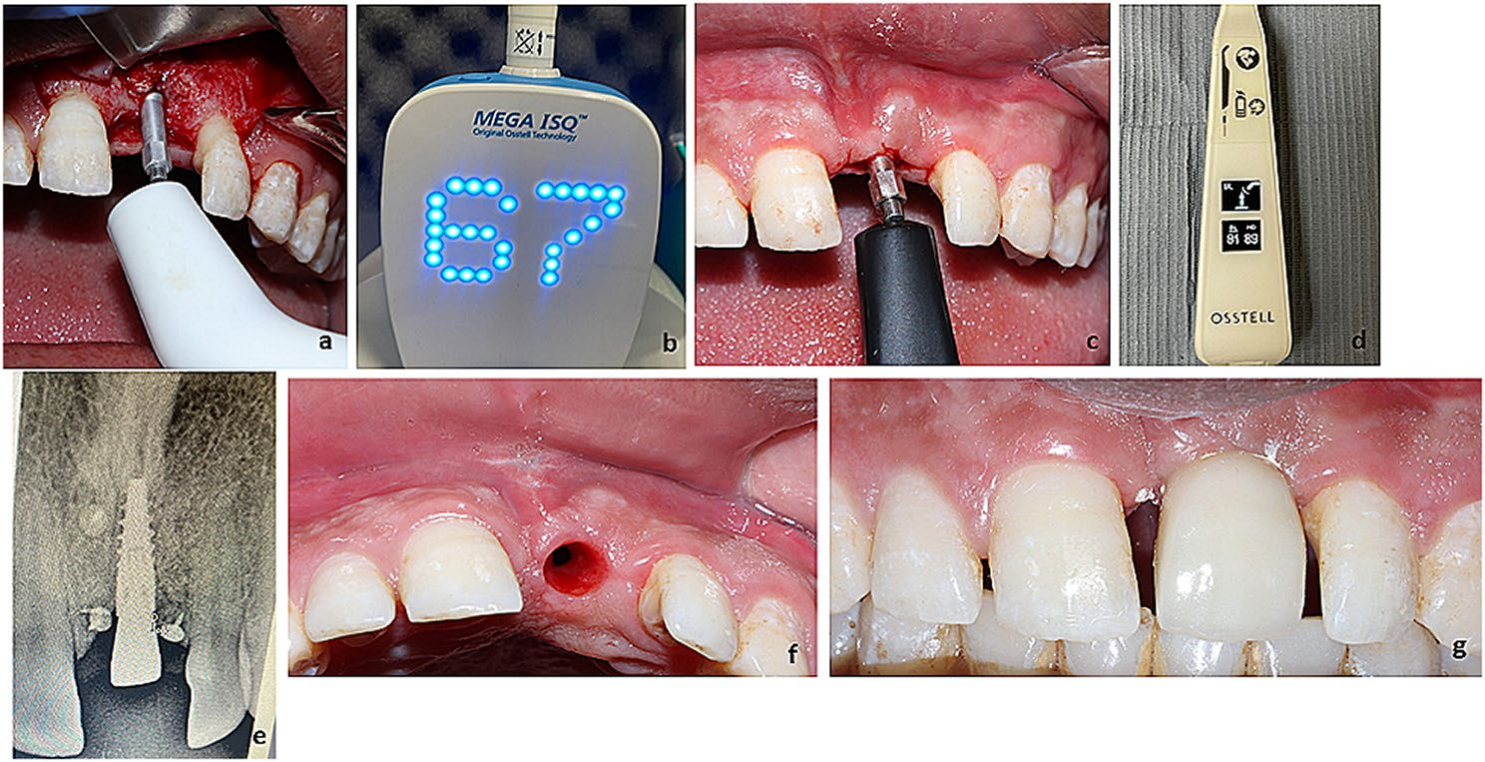
**a, b.** Primary implant stability measured after insertion (6 months from augmentation) that was 67 ISQ, **c, d**: secondary implant stability (6 months from implant placement) that was 89 ISQ, **e**: periapical radiograph of implant, **f**: implant uncovering showing a healthy pink gingival cuff, **g**: frontal view of final restoration of tooth immediately after cementation

**Fig. 3:**
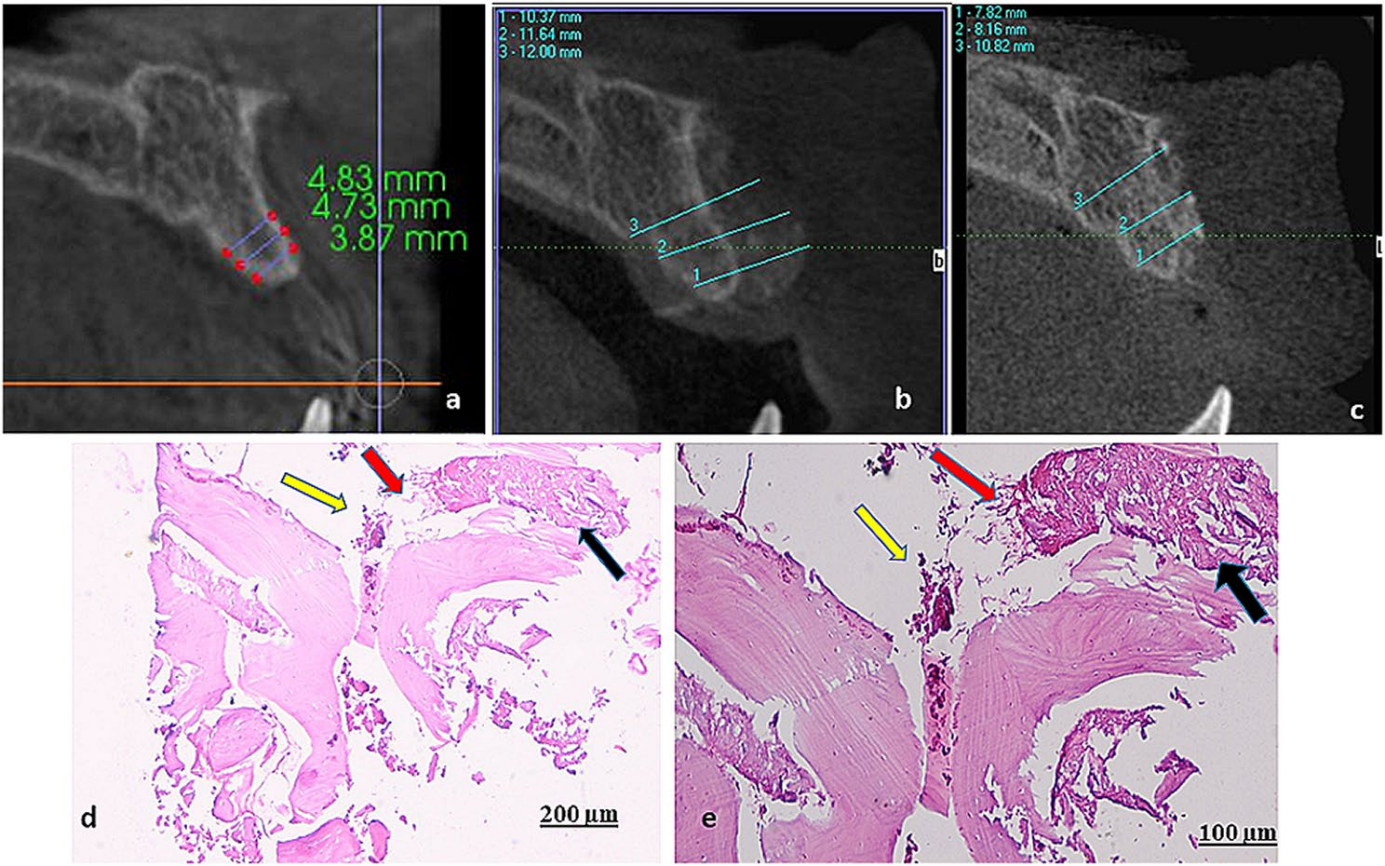
**a**. Preoperative CBCT measurements, **b**: CBCT measurements immediately after augmentation, **c**: CBCT 6 months after augmentation, **d**, **e**: Photomicrographs of H and E stained sections with different magnification of double layer technique (DLT), showing a considerable amount of newly formed bone (black arrow), little amount of fibrous tissue (red arrows) and residual graft (yellow arrows) (orig. mag. x10, x20)

The clinical results showed the healing was uneventful with no exposure, no signs of primary or secondary wound dehiscence. Clinically width of the grafted alveolar ridge was noted using the DLT and implant placed at the grafted sites showed primary stability that was assisted immediately after implant placement (67 ISQ) (Fig. 2a, b), 4 months following implant placement, the implant was successfully osteintegrated and fully loaded with secondary stability was (89 ISQ) (Fig. 2c, d). The radiographic images of the target areas revealed a homogeneous density of the augmented bone and alveolar bone. There was a significant gain of the buccolingual width that showed an increase from 3.87 mm preoperatively to 11.07 mm immediately postoperative and 7.87 mm 24 weeks postoperatively (Fig. 3a-c). Histological evaluation of H&E-stained biopsy sections revealed considerable amount of newly formed vital bone, these bone trabeculae were thicker, more organized. A little amount of both fibrous tissue and residual xenograft were revealed (Fig. 3d, e).

## Discussion

To obtain ideal osseointegration, an esthetic and fuctional accepted restoration, the maintenance of at least 1 mm of alveolar bone width in the buccal and palatal plane is required [19]. Insufficient bone volume does not allow correct and prosthodontically guided positioning of dental implants. The aim of the present case report is to evaluate the efficacy of the DLT for horizontal alveolar ridge augmentation and also evaluate the clinical, radiographic and implant stability outcomes.

Autogenous bone and bone substitutes with different resorption rates were placed in layers to increase the dimensions of the atrophic alveolar ridges according to the pilot study by Batas et al. [17], thus achieving an improved healing and a prolonged maintenance of the regenerated hard tissues. The main difference that in this case report that the first layer was autogenous bone which is provid better osteogensis and oseinductive properties. This means that the fast-resorbing autograft laid under the slow-resorbing xenograft [20] enhances vital bone formation, so double layer technique could prove throught this clinical case report, it could be suggested that DLT could have an effect on the stability of implant, clinical and radiographic outcome.

For clinical finding was that the ridge width increased from 4.5 mm preoperatively to 11.5 mm immediately post-operative and reached to 8.5 mm 6 months post-operative with total amount of ridge width gain 4 mm. The average horizontal gain obtained via the GBR technique in this case report is consistent with previously published results regarding atrophic ridges treated with similar techniques (bone regeneration with particulate graft and resorbable membrane) which reported horizontal gains from 1.5 to 3.8 mm [21, 22]. There was bone loss from immediately postoperative 11.3 mm to 6 months postoperative 8.9 mm this according to the literature, sites augmented with mandibular bone have resorption rates between 5% and 28% [23].The radiographic ridge width increased from 4.4 mm preoperatively to 11.3 mm immediately post-operative and reached to 8.9 mm 6 months postoperative with total amount of radiographic ridge width gain 4.6 mm which was in accordance to previous studies using particulate allografts or xenografts (alone or in combination with particulate autogenous bone) reported a wide range of bone loss (0.54–3.1 mm) [24, 25]. Regarding the histological assessment, there was a high percentage of newly formed bone, with high quality and the continuity of the newly formed bone trabeculae with the native bone, also the layering technique yields formation of thicker, well-organized, relatively highly cellular trabeculae. These findings suggest better osteoinduction and formation of stronger bone augmented to the native bone, thus, able to withstand loading forces of the dental implant. The measurement of implant stability is implant stability quotient (ISQ) that ranges from 1 (lowest implant stability) to 100 (highest implant stability) [26, 27]. ISQ values for successful implants range from 57 to 82. However, ISQ values at implant insertion should be ≥ 60 to achieve sufficient implant stability, in our case report the primary implant stability was 67 ISQ and secondary implant stability after 6 months form implant placement was 81 ISQ. One of the limitation of this case report is further follow up regarding the survival rate and stability of implant with radiographic analysis for the crestal bone level.

## Conclusion

DLT was successfully used for horizontal alveolar ridge augmentation, thus allowing a prosthetically driven implant placement. More cases assessing implant survival and success are needed to confirm the results of this case report.

## Data Availability

The data sets used and analyzed during the current study are available from the correspondingauthor on reasonable request.

## Footnotes

1 ωNeobiotech, Seoul, Korea.

2 ∝Jason, Botiss, Germany.

3 θTitan, Botiss, Germany.

4 ρ Bio-Oss ®, Geistlich Pharma, Wolhusen, Switzerland.

5 υ Polypropylene blue, Assut medical, Switzerland.

6 ∋ GlaxoSmithKline, Egypt.

7 ϕ Flagyl, Sanofi Aventis, Egypt.

8 τ Alphintern; Amoun, Egypt.

9 ν Brufen, Kahira Pharmaceuticals, Egypt.

10 ζ Antiseptol; Kahira pharm, Egypt.

11 ϖ smithcare trephine bur.

12 η JDEvolution S, a two-piece implant, Italy.

14 Δ Imaging Sciences International, Hatfield, Pa.
